# Emission Controls Using Different Temperatures of Combustion Air

**DOI:** 10.1155/2014/487549

**Published:** 2014-05-21

**Authors:** Radovan Nosek, Michal Holubčík, Štefan Papučík

**Affiliations:** University of Zilina, Univerzitna 8215/1, 010 26 Zilina, Slovakia

## Abstract

The effort of many manufacturers of heat sources is to achieve the maximum efficiency of energy transformation chemically bound in the fuel to heat. Therefore, it is necessary to streamline the combustion process and minimize the formation of emission during combustion. The paper presents an analysis of the combustion air temperature to the heat performance and emission parameters of burning biomass. In the second part of the paper the impact of different dendromass on formation of emissions in small heat source is evaluated. The measured results show that the regulation of the temperature of the combustion air has an effect on concentration of emissions from the combustion of biomass.

## 1. Introduction

The main intention of European Union is to exploit the potential of energy savings and renewable sources. In Slovakia the most promising renewable energy source seems to be biomass. Its use has growing importance. The most common form of biomass is wood, either in pieces or as wood waste. During the combustion process of renewable fuels pollutants are generated into the atmosphere and have a negative impact on human health. The most monitored pollutants are particulate matter, carbon monoxide, nitrogen oxides, and sulphur dioxide [1, 2].

Emissions emitted during combustion are mainly constituted of gaseous and particulate pollutants. The aim is to reduce the concentration of these substances to acceptable levels, since the emissions have a significant proportion of air pollution [3].

The solid particles are entrained with flue gas stream from the combustion chamber of boiler. Particulate matter (PM) consists of soot, inorganic matter (ash), and organic matter (nonvolatile flammable). Particles are imported into the flue gas by ash, nonvolatile, and combustible soot.

Particulate matter formation during fuel combustion depends on many factors, including flame temperature, composition and concentration of combustion reactants, and residence time within the reaction zone [4]. Although PM formation from combustion is not fully understood, it is suspected that the process involves both nucleation and condensation mechanisms [5].

The size of particles formed during combustion is dependent on the time spent in the formation and oxidation zones. The size of a biomass exhaust particle can span a range from less than 0.01 *μ*m to greater than 100 *μ*m. However, the majority of biomass combustion aerosol is typically smaller than 1 *μ*m in diameter [6].

Today is the greatest attention paid to the size of particles (aerodynamic diameter) less than 10 *μ*m (PM10), which may penetrate into the respiratory tract. Particles of this fraction are divided into two groups based on different sizes, the mechanism, the composition, and behaviour of the atmosphere.

The first group is made up of particles of size below 2.5 *μ*m (fine respirable fraction—PM2,5), arising from chemical reactions nucleation, condensation of gaseous emissions generated at the surface of particles, or coagulation of the finest particles.

The second group created particles in the range of the size from 2.5 to 10 *μ*m (coarse fraction—PM2,5 to 10).

Finest particles with a diameter below 2.5 *μ*m (PM2,5) are considered to cause the greatest harm to human health. They deposit deep in the lungs and block the reproduction of cells [7–9].

Various types of wood have different composition and properties such as calorific value and ash melting behavior of temperature, which greatly affect the production of PM.

In this work, experimental measurements were carried out and focused on the formation of PM during combustion of different types of dendromass in a small heat source. The effect of various temperatures of the primary combustion air to the emission parameters is also evaluated.

## 2. Measurement of Emission Parameters

Methods for measuring emissions of pollutants can be divided in principle into measuring of particulate matter and gaseous substances. Methods and measurement principles are based on the emission properties of the fluid medium. One of the method for measuring particulate matters is presented below.

*Gravimetric Method*. Gravimetric method is the manual single method with sampling of the flow gas by probe. It is based on determination of the median concentrations by sampling from multiple points of measurements cross-section and their subsequent gravimetric assessment. Solid contaminants are usually separated by an external filter.

Representative sampling is performed by sampling probe suitable shape and the correct speed under isokinetic condition [10].

Concentration of particulate matter in the flue gas is covered to standard conditions and can be determined for wet or dry flue gas. Measured volume of sample taken on the volume gas meter should be converted to standard conditions, that is, 101325 Pa pressure and temperature of 273.15 K (0°C). Therefore, the temperature and pressure of measured sample are measured before gas meter.

The cumulative collection can provide in the cross-section average concentration but not concentration profile. Flow velocity or flow of the sample gas is measured by ensuring of isokinetic conditions, for example, by aperture track and a total collected amount of gas by gas meter [11, 12].

In gravimetric method, the taking of representative samples is realized by probe with appropriate shape right from the flowing gas [13].

**Figure 1:**
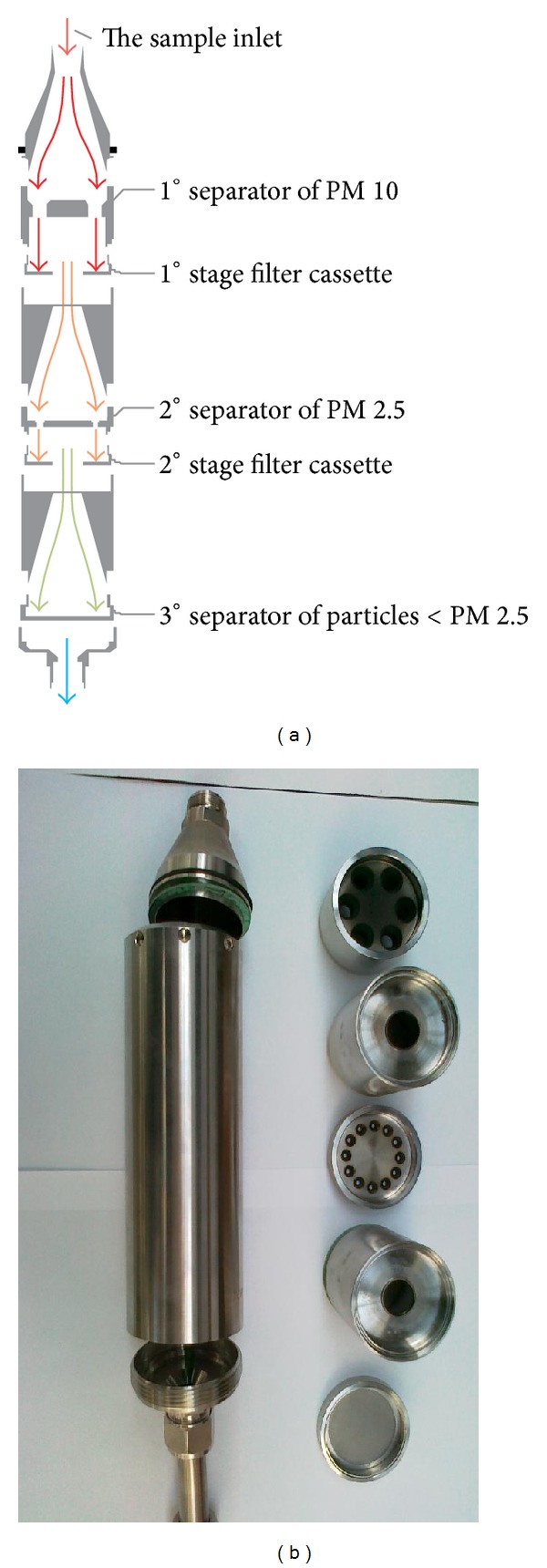
Multistage separation impactor.

To meet the increasing requirements toward the fine particulate determination, the multistage impactor probe was used in these experiments. Impactor separation system is intended to filtrate and separate solid emissions in three-stage impactor. The construction of device allows parallel separation of solid elements PM 10 and PM 2,5 (Figure 1).

The advantage of the gravimetric method is its simplicity and the relatively low sampler costs.

## 3. Experimental Measurement

As the heat source was used fireplace rated at 6 kW, which is designed for burning of piece wood. Bottom of the combustion chamber is topped with grate and the container where the ash falls. Access to the combustion chamber is through the doors that are glazed with high heat resistant glass.

### 3.1. Cooling/Heating of Combustion Air

Changing the temperature of the combustion air inlet was performed on the primary combustion air. The heat exchangers are plugged to pipe of primary air supply for heating/cooling of combustion air. This way is the temperature of the incoming primary combustion air heated/cooled to the desired temperature level. The minimal supply air temperature was –5°C and gradually increased up to 40°C. The increase in temperature between the measurements was 5°C and was regulated by the heat exchanger, which is located behind the fan in a duct. Temperature control for the heat exchanger was ensured by circulatory thermostat Julabo F40.

**Figure 2:**
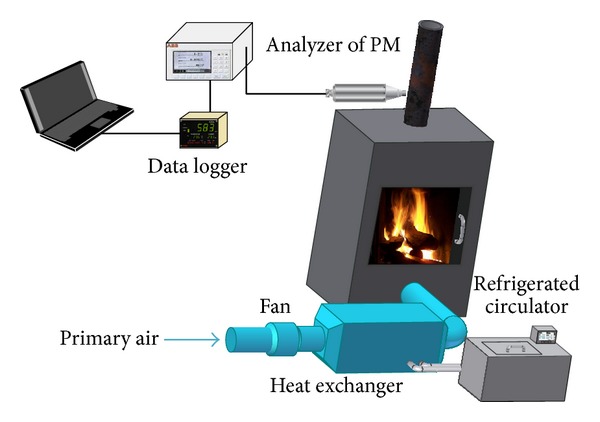
Scheme of experimental setup.

The scheme of experimental stand for the heating/cooling air supply is shown in Figure 2.

In order to evaluate the quality of combustion process, the gas composition was measured by analyzer.

### 3.2. Dendromass

During the experiment, the different types of wood were tested as well. Every measurement lasted 1 hour and was burned to about 1,5 kg of fuel. For the experimental measurements the following types of wood that are listed in Table 1 were used.

### 3.3. Position of Secondary Air

Modern modifications allow an increase of heating efficiency and reduction of emission concentration. The amount of emissions can be affected by several factors. One of the important factors is the position of secondary combustion air.

**Figure 3:**
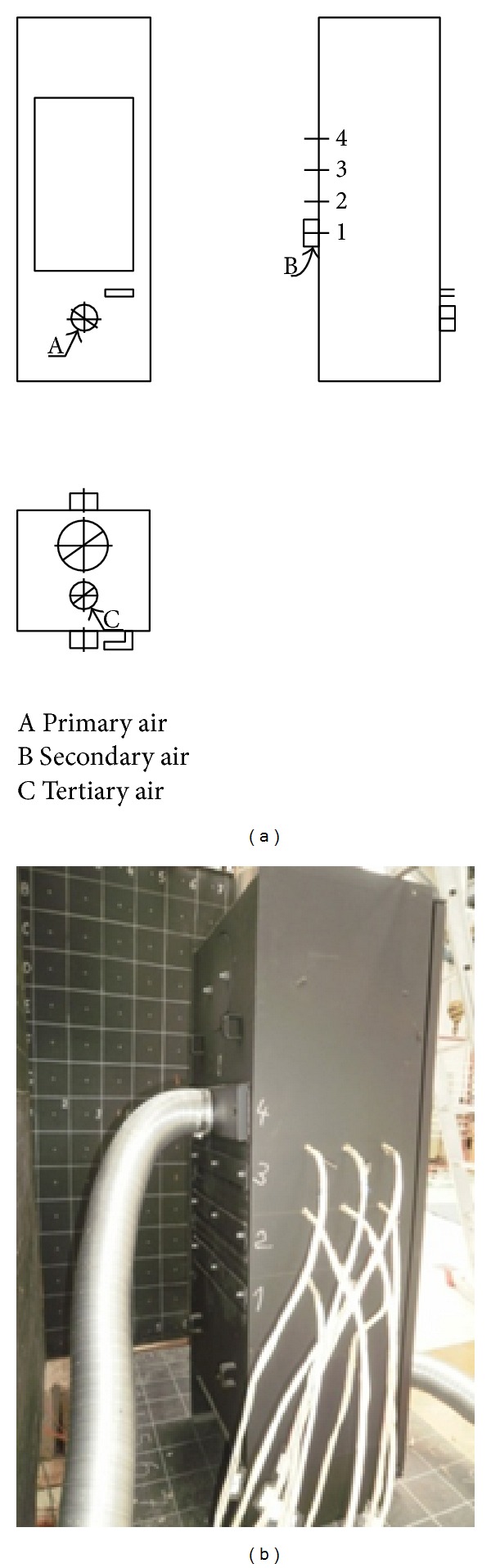
Position of combustion air inlets.

The experimental heat source has the following air inlets:

- primary (frontal)—airflow through the grate and ashtray towards fuel,
- secondary (back)—process using residual combustible gases that would normally escape through the chimney. There is an increase in efficiency and thus lower fuel consumption,
- tertiary (top)—used for blowing off the windshield, preventing clogging, also contributing to improvement of combustion process, and reducing emissions. Fireplace is designed for burning of piece wood (see Figure 3).

In this task, the different positions of secondary air inlet were investigated. The aim was to evaluate whenever the location of air inlet has influence on the formation of particulate matter.

## 4. Results and Discussion

During the measurements concentrations of following emissions were recorded: CO, CO_2_, and NO and particulate matters in the flue gas.

### 4.1. Effect of Air Temperature on Formation of Emission

The temperature of the primary combustion air supplied to fireplace varied by changing the setting temperature on the refrigerated circulator.

Different temperatures of the primary combustion air have impact on formation of gaseous emissions and particulate matter.

**Figure 4:**
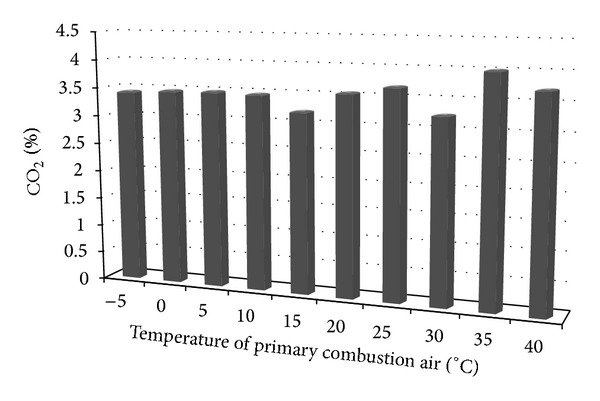
Average CO_2_ emissions depending on the temperature change of the primary combustion air.

Figure 4 shows the results of the measurement of carbon dioxide according to the set temperature of the primary combustion air.

The highest average CO_2_ was recorded at 35°C of inlet air, while at 15°C of supplied air the lowest average value of 3.20% was registered. Carbon dioxide formation has a trend to increase with increasing temperature of the primary combustion air.

**Figure 5:**
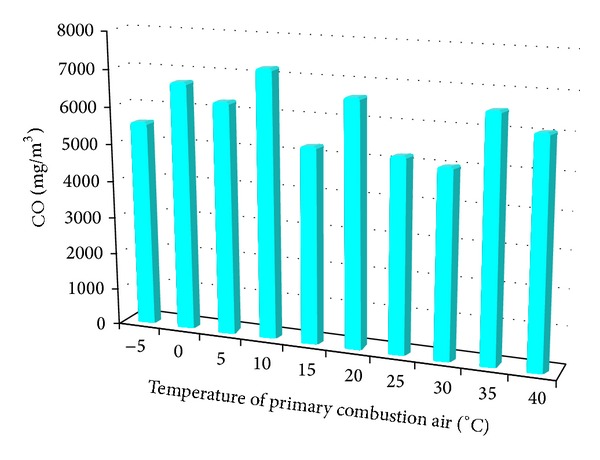
Average CO emissions depending on the temperature change of the primary combustion air.

Figure 5 shows the results of the measurement of carbon monoxide.

The highest average values reached 7193 mg·m^−3^ of CO and were recorded at 10°C inlet air, while at 30°C supply air reached the lowest average value of 5051 mg·m^−3^. The results indicate that formation of carbon monoxide has a trend to decrease with increasing temperature of the primary combustion air.

**Figure 6:**
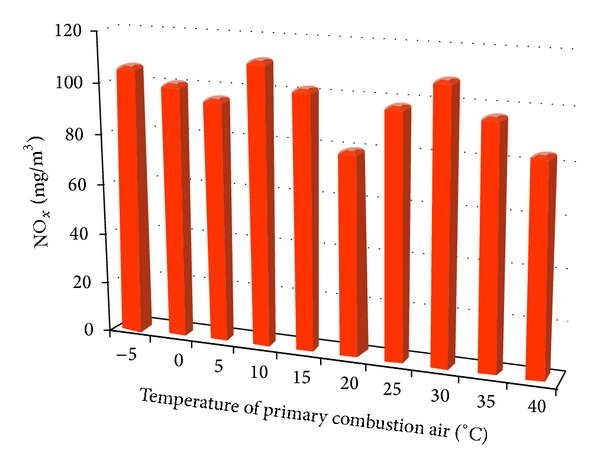
Average NO_*x*_ emissions depending on the temperature change of the primary combustion air.

Dependence of NO_*x*_ formation on the different temperatures of the primary combustion air to the experimental heat source shows Figure 6.

The highest average values of the measured NO_*x*_ (111.65 mg·m^−3^) were achieved at 10°C, and the lowest average values were measured at 20°C with a value of 80.16 mg·m^−3^. NO_*x*_ production has a trend to decrease with increasing temperature of the primary combustion air.

**Figure 7:**
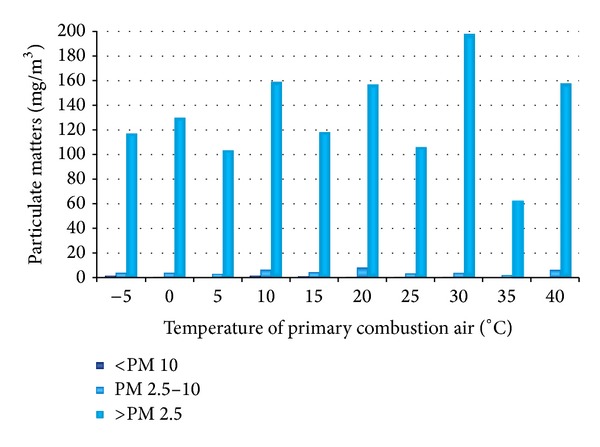
Concentrations of particulate matter for different temperatures of air.

**Figure 8:**
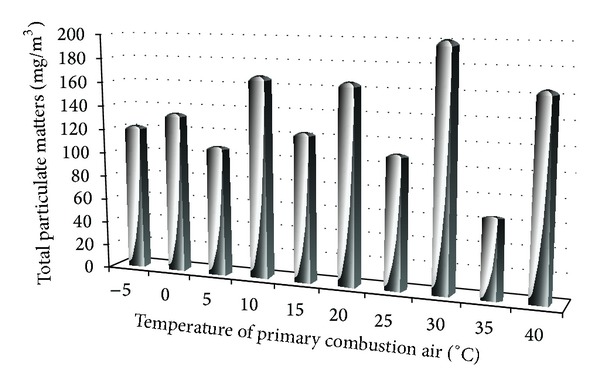
Dependence of total particulate matter on the temperature.

The results of PM concentration depending on the temperature of primary combustion air are shown in Figures 7 and 8.

Measurement of particulate matter with a change of temperature of combustion air has reached the maximum concentration of 202 mg·m^−3^. Minimum concentration of PM emission was generated at 35°C of combustion air.

### 4.2. Different Type of Dendromass

The second part of the work deals with the effect of different dendromass to formation of solid particles. Generation of emissions is largely influenced by type of fuel that is burned in heat source. Every fuel has different properties and chemical composition, which ultimately affects the combustion process, the amount of actual emissions, and ash content. During the experimental measurements the same combustion conditions were secured, that is, uniform supply of primary, secondary and tertiary air, the same pressure in chimney (12 Pa), and a maximum dose of 1.5 kg of fuel.

**Figure 9:**
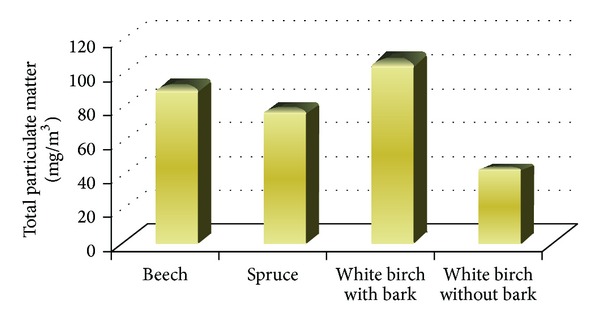
Concentrations of PM for different types of dendromass.

Particulate measurements were conducted on all types of wood for 30 minutes. During this time were captured PM to the filters from each sample. These were subsequently stripped of moisture and weighed. Concentrations of particulate matter were determined by difference weight of the filter before and after the measurement. The highest amount of particulate matter was observed in measurements of white birch with bark and beech (Figure 9).

### 4.3. Different Positions of Secondary Air

The final part presents the most effective location of secondary air inlet in relation to formation of particulate matters. The influence of three air inlets position was analyzed.

**Figure 10:**
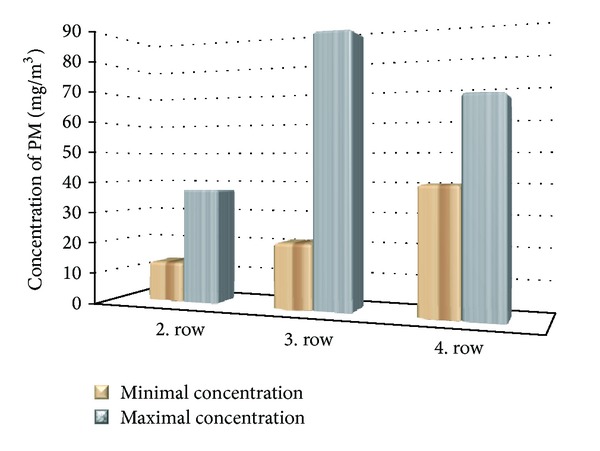
Dependence of PM on position of air inlet.

On Figure 10 the minimal and maximal values of measured particulate matter concentrations (PM) are shown. Measurement of PM for all fully open combustion air reached concentration 21 mg·m^−3^. Minimum concentration of PM was registered with the involvement of secondary supply in the second row, where only 13,09 mg·m^−3^ was measured.

It can be concluded that in terms of PM it is advantageous to supply the combustion air through second row.

## 5. Conclusion

The aim of this work was to demonstrate the impact of the primary combustion air temperature on emissions parameters.

Presented results of emissions depending on the temperature of the primary combustion air do not indicate the most suitable setting of temperature. For each type of emission the lowest value at different temperatures of the primary combustion air has been reached.

From the experimental measurements of solid emissions it is clear that in terms of the lowest value of PM it is preferred to supply the primary combustion air into the combustion process at a temperature of 35°C.

It can be argued that the production of carbon monoxide (CO) decreases with increasing temperature at the expense of higher production of carbon dioxide (CO_2_). The formation of CO is influenced by several factors and therefore its different concentration during the measurements cannot be attributed to changing temperatures of the combustion air.

In this research work analysis of the impact of different types of dendromass on the formation of particulate matters during the combustion process was carried out. The results of measurements indicate that the type of fuel has a considerable influence on the combustion process and the formation of particulate matters. This phenomenon is largely influenced by the different properties and chemical composition of different types of dendromass.

In the case of birch without bark, the lowest values of PM were measured, suggesting that the bark of firewood has a significant proportion on the formation of solid particles.

The measured results show that the type of firewood affects emission parameters of the heat source.

Computer modelling is becoming more powerful and developed, therefore gaining in popularity. It is emerging as an attractive tool to assist the combustion engineer in such areas as new process design, plant scale-up, retrofitting, and pollutant control. Therefore the numerical simulation of particulate matter formation will be done in the future research.

## Figures and Tables

**Table 1:** Types of wood and their parameters.

Type of fuel	Calorific value (MJ/kg)	Humidity (%)
Beech	17,5	7,64
Spruce	19,3	7,87
White birch with bark	28	18,15
White birch without bark	19	18,15
